# Molecules and Morphology Reveal Overlooked Populations of Two Presumed Extinct Australian Sea Snakes (*Aipysurus*: Hydrophiinae)

**DOI:** 10.1371/journal.pone.0115679

**Published:** 2015-02-11

**Authors:** Kate L. Sanders, Tina Schroeder, Michael L. Guinea, Arne R. Rasmussen

**Affiliations:** 1 School of Earth and Environmental Sciences, University of Adelaide, Adelaide, South Australia 5000, Australia; 2 Research Institute of the Environment and Livelihoods, Charles Darwin University, Darwin, Northern Territories 0909, Australia; 3 The Royal Danish Academy of Fine Arts, School of Architecture, Design and Conservation, Esplanaden 34, DK-1263, Copenhagen, Denmark; Simon Fraser University, CANADA

## Abstract

The critically endangered leaf-scaled (*Aipysurus foliosquamaI*) and short-nosed (*A. apraefrontalis*) sea snakes are currently recognised only from Ashmore and Hibernia reefs ~600km off the northwest Australian coast. Steep population declines in both species were documented over 15 years and neither has been sighted on dedicated surveys of Ashmore and Hibernia since 2001. We examine specimens of these species that were collected from coastal northwest Australian habitats up until 2010 (*A.foliosquama*) and 2012 (*A. apraefrontalis*) and were either overlooked or treated as vagrants in conservation assessments. Morphological variation and mitochondrial sequence data confirm the assignment of these coastal specimens to *A. foliosquama* (Barrow Island, and offshore from Port Hedland) and *A.apraefrontalis* (Exmouth Gulf, and offshore from Roebourne and Broome). Collection dates, and molecular and morphological variation between coastal and offshore specimens, suggest that the coastal specimens are not vagrants as previously suspected, but instead represent separate breeding populations. The newly recognised populations present another chance for leaf-scaled and short-nosed sea snakes, but coastal habitats in northwest Australia are widely threatened by infrastructure developments and sea snakes are presently omitted from environmental impact assessments for industry. Further studies are urgently needed to assess these species’ remaining distributions, population structure, and extent of occurrence in protected areas.

## Introduction

One in five reptile species might be at risk of extinction and many are thought to have become extinct within the last 50 years [1]. Threats to reptiles include habitat loss and degradation [1], climate warming [2], and overharvest for food, traditional medicines and leather [3]. Rediscoveries of presumed extinct species (e.g. [4]) inspire optimism, but many such rediscovered species remain at immediate risk of extinction and require urgent assessment of population status and threats in their remaining range to guide management actions[5, 6].

**Fig 1:**
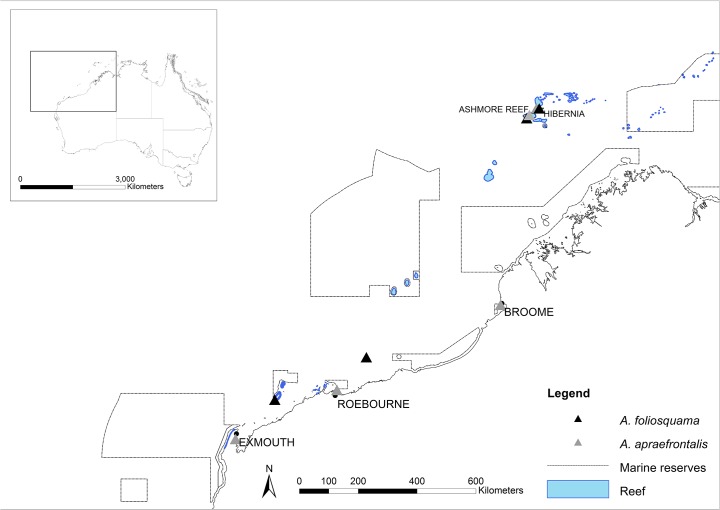
Map showing coastal and offshore localities for the *Aipysurus foliosquama* and *A*. *apraefrontalis* specimens included in this study. Also shown are the distribution of coral reefs and the Northwest Commonwealth Marine Reserves Network.

**Fig 2:**
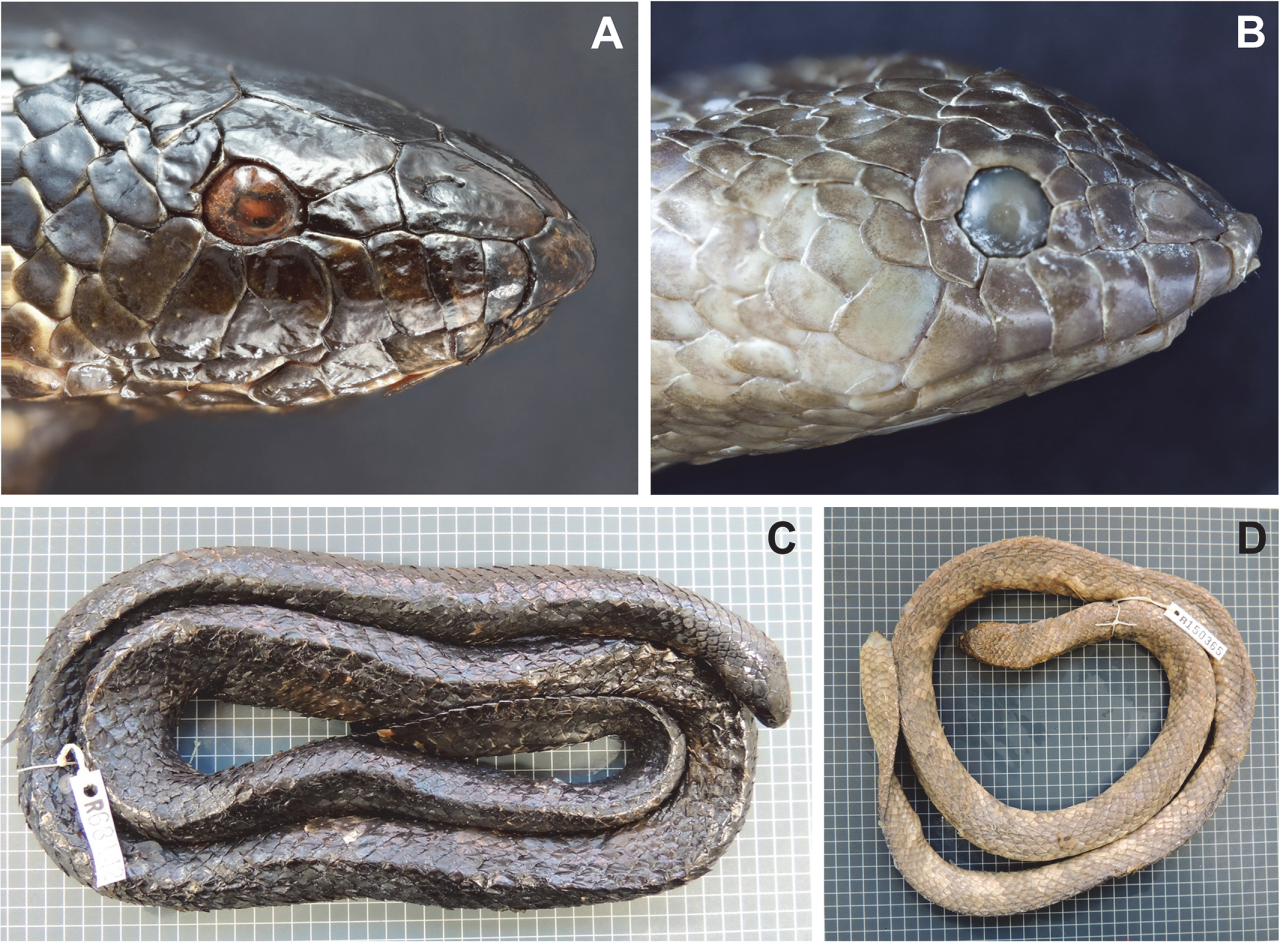
Photographs of A,C: *A. apraefrontalis* (SAMA R68142) from Ashmore Reef; and B, D: *Aipysurus foliosquama* (WAM R150365) from Barrow Island.

Viviparous sea snakes (Hydrophiinae) are the only extant group of fully marine reptiles, but remain very poorly understood with a recent IUCN Red List assessment listing 38% of species as data deficient [7,8]. Two species have been listed as critically endangered on the IUCN Red List and Australia’s EPBC Act (Environmental Protection and Biodiversity Act, 1999) due to their restricted distributions and documented population declines [7, 9, 10]. *Aipysurus foliosquama* (the leaf-scaled sea snake) and *A*. *apraefrontalis* (short-nosed sea snake) are currently recognised only from Ashmore and Hibernia reefs ~600km off the northwest Australian coast [11, 12, 13, 14] (Figs. 1 and 2). Surveys of these reefs undertaken in 1974 [11], between 1994 and 2007, and in 2012 and 2013 [15, 16, 17] indicated population declines of at least 90% in *A*. *foliosquama* and *A*. *apraefrontalis* over 15 years (three generation lengths). Neither species has been sighted on surveys of Ashmore and Hibernia (or any other Timor Sea reefs [18]) since 2001 despite dedicated search efforts in 2005, 2007, 2012 and 2013 [17]. However, in 1982 a single specimen identified as *A*. *foliosquama* was collected offshore from Port Hedland, and in 2010 a beach-washed specimen also identified as *A*. *foliosquama* was collected on Barrow Island. These localities are close to the northwest Australian coast, approximately 800km from Ashmore and Hibernia (Fig. 1). *Aipysurus apraefrontalis* has also been recorded from the northwest Australian coast: in the Exmouth Gulf and offshore from Roebourne and Broome up until 2012 (this study and [12]) (Fig. 1) and from the Arafura Sea [19]. These scattered specimens are searchable on museum collection databases (e.g. Atlas of Living Australia website at http://www.ala.org.au) but were treated as vagrants in the IUCN Red List and EPBC Act assessments on the assumption that breeding populations of both species are restricted to Ashmore and Hibernia [7, 9, 10]. Sea snakes are vulnerable to dispersal in strong currents during storms and locality records for many species include remote outliers.

Here, we examine morphology and mitochondrial sequences for the previously overlooked coastal specimens and *A*. *foliosquama* and *A*. *apraefrontalis* from Ashmore and Hibernia. These data verify the assignment of the coastal specimens to *A*. *foliosquama* (Barrow Island, and offshore from Port Hedland) and *A.apraefrontalis* (Exmouth Gulf, and offshore from Roebourne and Broome). Molecular and morphological variation between coastal and offshore specimens, in addition to their collection dates, indicate that the coastal specimens are not vagrants but instead represent overlooked breeding populations. We discuss conservation implications and provide revised accounts of the two species’ internal and external morphology, geographic distributions and ecology.

## Material and Methods

### Molecular analyses

We sampled six specimens of *Aipysurus foliosquama*: five from Ashmore Reef collected between 1996 and 1998 (SAMA R68139, SAMA R68145, SAMA R68144, SAMA R68146, SAMA R68143), and the specimen collected on Barrow Island in 2010 (WAM R150365). DNA tissues are not available for the other coastal *A*. *foliosquama* (from offshore Port Hedland). Four *A*. *apraefrontalis* were sampled: one collected on Ashmore in 1996 (SAMA R68142), two collected in the Exmouth Gulf in 2000 and 2012 (voucher specimens held in the Western Australian Museum: WAM R157818, WAM R154750) and one collected in 2012 on Cable Beach near Broome (WAM R174539). To our knowledge, no additional DNA tissues are available for coastal *A*. *apraefrontalis*. Because our study provides the first molecular data published for *A*. *foliosquama* we tested a sister species relationship between *A*. *foliosquama* and *A*. *apraefrontalis* by additionally sampling *A*. *duboisii* (recovered as the sister lineage to *A*. *apraefrontalis* in a phylogenetic analysis of five nuclear loci that included those two species but not *A*. *foliosquama*: [20]). Of the four *A*. *duboisii* sampled, one was from the Exmouth Gulf (WAM R154751), two were from Hibernia (NTMR17781, NTMR17785) and one was from Seringapatam Reef (no voucher specimen). Samples collected by the authors were obtained by snorkelling with nets (Ashmore, Seringapatam) or from beach-washed specimens (Broome) under Western Australia Department of Parks and Wildlife (DPaW) scientific permits, and animal ethics approval from the University of Adelaide Animal Ethics Committee (approval number 000018247) and Charles Darwin University Animal Ethics Committee. No endangered species were sacrificed during our study. Sampling localities are shown in Fig. 1.

Standard Proteinase K protocols were used to extract whole genomic DNA from tissues (liver biopsies and tail clippings) and fragments were amplified using HotMaster Taq polymerase reagents (Perkin Elmer). The double-stranded amplification products were visualised on 1.5% agarose gel. We sequenced 1100 bp of the mitochondrial cytb (cytochrome b) gene. Primers were Forward L14910 (5’- GAC CTG TGA TMT GAA AAACCA YCG TTG T -3′) and Reverse H16064 (5’- CTT TGG TTT ACA AGA ACA ATG CTT TA -3’) [21]. Annealing temperatures ranged between 55 and 59°C. Double-stranded sequencing was outsourced to the Australian Genome Research Facility Ltd (AGRF) in Adelaide, Australia.

Sequences were checked for ambiguities, and alignments were assembled from consensus sequences of forward and reverse reads using Geneious Pro v5.1.7 software [22]. Polymorphism and divergence statistics were calculated using DnaSP v5 [23] and the Species Delimitation plugin for Geneious Pro [24]. Phylogenetic analysis used Bayesian inference implemented in BEAST 1.8.0 [25]. Various partitioning strategies and clock and substitution models were trialled during preliminary runs. Optimum convergence was achieved using the final analysis settings of: unlinked partitions of 1st + 2nd codon positions (GTRig) and 3rd positions (GTRig), a Yule tree model prior with a uniform distribution, and a strict clock model implying no branch rate variation. This analysis was run for 100,000,000 MCMC generations and sampled every 10,000 generations. Convergence was assessed in Tracer 1.4 [26] by examining likelihood plots and histograms and effective sample sizes (ESS values) of the estimated parameters. The first 3000 sampled trees were excluded as burn-in and the remaining 7,000 were used to generate a maximum credibility tree with Tree Annotator 1.7.1 [25].

### Morphological analyses

We examined 26 *Aipysurus foliosquama* from Ashmore and the two specimens from Barrow Island and Port Hedland; and 17 specimens of *A*. *apraefrontalis*: seven from Ashmore, six from the Exmouth Gulf, two from Roebourne and two from Broome. Two other coastal specimens provisionally identified as *A*. *apraefrontalis* are accessioned in the Western Australian Museum but were not available for study at the time of writing. Measurements and scale counts follow Smith [27] with the modification that scale rows were counted directly around the body (see [28]). The absolute position of the posterior tip of the heart and liver were determined in relation to the count of the adjacent ventral scales (VS), and their relative position is expressed as the percentage of the number (counted from head to rear) of the underlying ventral scale (%VS-heart, %VS-liver) (e.g. [29, 30]). Body length was measured from snout to vent and tail length from vent to tip of the tail.

Institutional abbreviations follow [31]: AMS = Australian Museum Sydney; BMNH = Natural History Museum London; NMNL = National *Museum of Natural History* Naturalis, *Leiden*; QM = Queensland Museum, Australia; SAMA = South Australian Museum; WAM = Western Australian Museum.

Specimens examined:

*Aipysurus foliosquama*: AMS R37143, AMS R40488, AMS R40489, AMS R40496, AMS R41516, AMS R41526, AMS R41537, AMS R44456, AMS R44576, AMS R44592, AMS R44597, AMS R44619, AMS R44872, AMS R44890, AMS R44896, AMS R44899, AMS R44902, AMS R44906, BMNH 1926.2.16.7 (holotype), NMNL 6430, SAMA R68139, SAMA R68145, SAMA R68144, SAMA R68146, SAMA R68143, WAM R129806 (all Ashmore Reef). AMS R104810 (offshore from Port Hedland).WAM R150365 (Bandicoot Bay, Barrow Island).

*Aipysurus apraefrontalis*: AMS R44616, AMS R44878, BMNH 1946.1.1.94 (holotype), BMNH 1946.1.1.95, BMNH 1926.5.28.21-22, SAMA R68142 (all Ashmore Reef). WAM R26415, WAM R31443, WAM R41261, WAM R43897, WAM R47748, WAM R157818 (all Exmouth Gulf). WAM R22961, QM J80569 (offshore from Roebourne).WAM R26716 (offshore from Broome), WAM R174539 (Cable Beach, Broome).

## Results

### Molecular analyses

**Fig 3:**
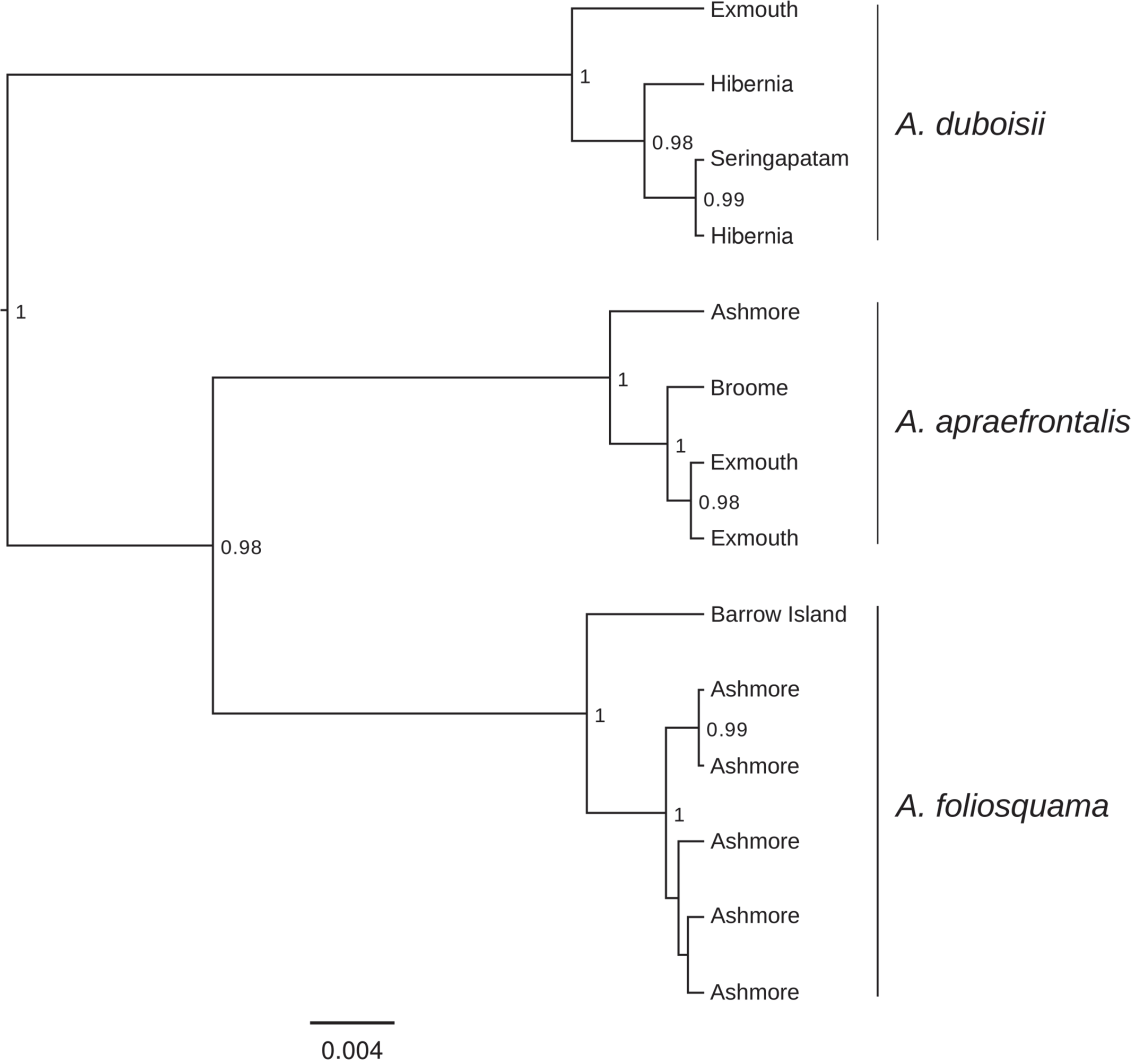
BEAST maximum clade credibility tree for the mitochondrial cytochrome b gene. Node support values (posterior probabilities) above 0.95 are shown. Scale bar indicates the number of substitutions per site.

The final data matrix comprised 14 individuals of the three *Aipysurus* species and 1011 base pairs. Sequence data are deposited at GenBank (accession numbers KP205504-KP205513). Translation of the protein-coding cytb gene did not reveal frameshifts, unexpected stop codons or indels. Bayesian analyses yielded effective sample sizes (ESS values) well above 1000 for all parameters. The maximum clade credibility tree recovered *Aipysurus foliosquama* and *A*. *apraefrontalis* as reciprocally monophyletic sister species with a posterior probability (pp) of 0.98 (Fig. 3), and these formed a clade with *A*. *duboisii* that was supported by a pp of 1.0. The mean pairwise distance (HKY) between *A*. *foliosquama* and *A*. *apraefrontalis* was 4.3%, and these species showed 5.4–5.9% pairwise divergence from *A*. *duboisii*. Coastal samples of *A*. *foliosquama* and *A*. *apraefrontalis* represented unique haplotypes that formed strongly supported sister lineages to conspecifics from Ashmore (pp 1.0). Intraspecific pairwise distances were 0.7% between *A*. *apraefrontalis* from Ashmore and Exmouth/Broome, 1.0% between *A*. *foliosquama* from Ashmore and Barrow Island, and 1.2% between *A*. *duboisii* from Exmouth and Hibernia/Seringapatam.

### Morphological analyses

Consistent with the molecular results, our morphological analyses clearly verified the identification of the Barrow Island and Port Hedland specimens to *A*. *foliosquama* (Table 1) and the remaining coastal specimens (Exmouth, Roebourne, Broome) to *A*. *apraefrontalis* (Table 2). We also found several differences between coastal and offshore specimens of both species: the two coastal specimens of *A*. *foliosquama* had higher ventral counts and more posterior relative heart positions than the other females examined from Ashmore (Table 1); coastal male *A*. *apraefrontalis* had higher ventral counts that did not overlap with conspecifics from Ashmore (Table 2) and reduced head relative to body size (not shown).

**Table 1:** Morphological characters for *Aipysurus foliosquama* from Ashmore Reef compared with the two coastal specimens.

Character	Locality (# specimens)		
	Ashmore M (18)	Ashmore F (8)	Coast F (2)
Ventrals	146–154	139–148	153–154
Subcaudals	23–27	19–22 (27*)	22–24
“Loreal”	yes	Yes	yes
Supralabials	7–8	7–8	7
Body scale rows	19	19	19
Neck scale rows	19	19	19
Deep ventral notch	yes	Yes	yes
VS-Heart	36–42	40–44	42–43
%VS-Heart	24.3–28.4	28.2–29.4	25.9–27.4

Data: own observation.

*One paratype with 27 subcaudals, for more information see “External Morphological Characters”. See Materials and Methods for character descriptions and abbreviations.

**Table 2:** Morphological characters for *Aipysurus apraefrontalis* specimens from Ashmore Reef compared with specimens from the Australian northwest coast.

Character	Locality (# specimens)			
	Ashmore M (6)	Ashmore F (3)	Coast M (7)	CoastF (2)
Ventrals	141–148	148–151	150–158	148–152
Subcaudals	19–25	19–25	22–27	19–23
“Loreal”	No	No	no	no
Supralabials	6–7	7	6–8	7
Body scale rows	17	17	17	17
Neck scale rows	17–19	17–19	17–19	17–19
Deep ventral notch	Yes	Yes	yes	yes
VS-Heart	38–42	41(n = 1)	40–47	42 (n = 1)
%VS-Heart	26.0–29.4	27.7 (1)	26.7–29.7	27.6 (1)

Data: own observation. See Materials and Methods for character descriptions and abbreviations.

### Species accounts

***Aipysurus foliosquama*** Smith, 1926.

*Aipysurus foliosquama* Smith, 1926 [8, 11, 12, 14, 16, 27, 32, 33, 34, 35, 36, 37, 38, 39, 40, 41, 42, 43, 44, 45, 46, 47, 48].

Synonyms

*Aipysurus (Smithohydrophis) foliosquama* [49, 50, 51]

*Smithohydrophis foliosquama* [52]

Diagnostic Features

*External Morphological Characters*: Teeth: 5–8 very small maxillary teeth behind poison fang, 5–7 palatine teeth, 22–23 pterygoid teeth, 15–16 dentary teeth. Scales: “Loreal” scale present;1–2 pre- and 2–3 postoculars; 7–8 supralabials; supralabials 1 and 2 invariably in contact with nasal, 3 and 4 or only 4 in contact with preocular, 4 and 5 or only 4 invariably in contact with eye; 2–3 anterior temporals; 7–9 infralabials; infralabials 1, 2 (sometimes divided), 3 and 4 in contact with anterior pair of sublinguals; infralabials 4 or none touching posterior pair of sublinguals. 19 scale rows on neck in males, 19 in females; 19 scale rows on body in males, 19 in females. 146–154 ventrals in males, 139–154 in females. Ventrals with a well-marked median keel and a deep notch on the posterior border. 23–27 subcaudals in males, usually 19–24 in females (but 29 in one paratype counted by Smith [25], however, we counted 27 subcaudals in the same specimen). Snout-vent length 80 cm in largest males, 95 cm in largest females. Tail length 10 cm in largest male, 11.5 cm in largest female.

*Internal Morphological Characters*: Tip of heart extending to ventral scale 36–42 in males, 40–44 in females. % VS heart 24.3–28.4 in males, 27.4–29.7 in females. Anterior end of liver situated at ventral scale 42–50 in males, 44–50 in females. %VS liver 28.3–33.6 in males, 28.7–33.7 in females. Heart and liver separated by 5–10 ventral scales in males, 3–6 in females. Body vertebrae: *Aipysurus foliosquama* shows a one to one correspondence between ventral body scales and vertebrae giving the same counts as heart and liver position in relation to the ventrals.

*Colouration*: Head brown above, same or lighter below. Body uniform tan-brown or purplish-brown, sometimes with whitish spots or bands on the sides and ventrally. Scales sometimes with dark margins. A pregnant female contained two large embryos, one with whitish bands around the body, the other with whitish bands only on the sides and ventrally.

*Geographical Distribution*: *Aipysurus foliosquama* is known from breeding populations at Ashmore and Hibernia Reefs in the Timor Sea [11,14, 16, 27, 32, 38, 45] that are now presumed extinct. The only other records are two specimens collected from Barrow Island and offshore from Port Hedland close to the northwest Australian coast (this study, Fig. 1).The type specimen was collected from Ashmore Reef [27].

*Habitat and Biology*: At Ashmore *Aipysurus foliosquama* has been found in shallow water not deeper than 10m, preferring areas heavily grown with corals [11, 16, 27, 32, 45]. Four prey species have been found in stomach contents of the Ashmore population. McCosker [53] reported jaws and a neurocranium identified to the family Labridae, presumably from a species of *Halichoeres*. Smith [27] reported that three specimens contained items identified as *Platyglossus* (= *Halichoeres*) *trimaculatus*. Voris [53] observed two specimens of the family Eleotridae and one specimen of the family Clinidae (Tripterygion). One pregnant female collected in January 1973 from Ashmore (AMS R44899) contained two full-term female embryos measuring 27 cm and 26.5 cm from head to vent.

***Aipysurus apraefrontalis*** Smith, 1926.

*Aipysurus apraefrontalis* Smith, 1926 [8, 11, 12, 14, 16, 19, 27, 32, 33, 34, 35, 36, 37, 38, 39, 40, 41, 42, 43, 44, 45, 46, 47, 48].

Synonyms

*Aipysurus (Smithohydrophis) apraefrontalis* [49, 50, 51].

*Smithohydrophis apraefrontalis* [52].

Diagnostic Features

*External Morphological Characters*: Teeth: 5–6 maxillary teeth behind poison fang, 7–8 palatine teeth, 17–19 pterygoid teeth, 16dentary teeth. Scales: “Loreal” scale absent; 1 pre- and 2 postoculars; 6–8 supralabials; supralabials 1, 2 and 3 in contact with nasal, 3 and 4 in contact with preocular, 4 and 5 or 4, 5 and 6 in contact with eye, sometimes superlabial 5 divided horizontally; 1–3 anterior temporals. Prefrontal scales missing. 6–8 infralabials; infralabials 1, 2 and 3 in contact with anterior pair of sublinguals, sometimes only 1 and 2 or 1, 2 and 4; posterior sublinguals separated. Both pairs of sublinguals small. 17–19 scale rows on neck in males, 17–19 in females; 17 scale rows on body in males, 17 in females. Scales smooth or with a tubercle or short keel.141–158 ventrals in males, 148–152 in females. Ventrals large, at least three times as large as adjacent body scales. Each ventral scales with a deep notched and a median keel. 19–27 subcaudals in males, 19–25 in females. Snout-vent length 98 cm in largest males, 94 cm in largest females. Tail length 10.5 cm in largest male, 9 cm in largest female.

*Internal Morphological Characters*: Tip of heart extending to ventral scale 38–47 in males, 41–42 in females. % VS heart 26.0–29.7 in males, 27.6–27.7 in females. Anterior end of liver situated at ventral scale 45–47 in males, 47 in females. %VS liver 30.8–32.9 in males, 31.76 in females. Heart and liver separated by 5–7 ventral scales in males, 6 in females. Body vertebrae: *Aipysurus apraefrontalis* shows a one to one correspondence between ventral body scales and vertebrae giving the same counts as heart and liver position in relation to the ventrals.

*Colouration*: Head brown, body tan to dark brown or purplish-brown, paler ventrally, with cream or whitish scattered scales or/and with lighter brown cross-bands, which are mostly confined to the lower parts of the body. The posterior portion of the body scales often darker than the anterior.

*Geographical Distribution*: *Aipysurus apraefrontalis* has been collected from Ashmore and Hibernia Reefs but is absent from the other coral reefs of the Timor Sea (Cartier Island, Scott and Seringapatam Reefs). Specimens collected from the Exmouth Gulf, Roebourne and Broome (this study and [12]) were assigned to *A*. *apraefrontalis* (this study). Shuntov [19] collected *A*. *apraefrontalis* from the Arafura Sea but provided no further information. Golay *et al*. [36] mentioned this species as occurring in Javan waters (Indonesia), but we could not confirm this record with any specimens. *Aipysurus apraefrontalis* is then only known from Western Australia [11, 16, 27, 38, 45], but may be extinct or near-extinct on the Timor Sea reefs [17]. The type specimen was collected from Ashmore Reef [27].

*Habitat and Biology*: At Ashmore *Aipysurus apraefrontalis* seems to be confined to shallow water at depths no greater than 10m [11]. It has been reported primarily from reef flat and reef edge habitats but compared to *A*. *foliosquama* seems to prefer sandy bottom habitats less heavily grown with coral [11, 32]. Some specimens were found in cavities under dead coral debris fully exposed at low tide [32] or resting beneath small coral overhangs or coral heads in 1–2 m of water [53]. Specimens from the Exmouth Gulf were collected in prawn trawl nets, presumably from inter-reef habitats [12].

Stomach contents data are available only for the Ashmore population, with one specimen reported to contain a ‘small eel’ [27] and two specimens containing gobies (Eleotridae) [54]. Two male specimens collected in the Exmouth Gulf in late June 1973 and July 2004 had very rugous ventral scales indicating breeding condition; a single gravid female was collected in Exmouth Gulf in late November 1967.

## Discussion

Our molecular and morphological data confirm the assignment of coastal northwest Australian specimens to *Aipysurus foliosquama* (Barrow Island, and offshore from Port Hedland) and *A*. *apraefrontalis* (Exmouth Gulf, and offshore from Roebourne and Broome). For both species, coastal specimens represent moderately divergent mitochondrial haplotypes not present in sampled conspecifics from Ashmore. Morphological differences are also found between coastal and Ashmore/Hibernia specimens: The two coastal *A*. *foliosquama* females have more ventral scales and more posterior relative heart positions than conspecific females from Ashmore (Table 1); coastal *A*. *apraefrontalis* have more ventrals in males (Table 2) and consistently smaller heads relative to body size compared to Ashmore specimens (not shown). These molecular and morphological differences suggest that the coastal specimens are not vagrants as previously suspected, but instead represent separate breeding populations. Collection dates of the coastal specimens further support this interpretation: *A*. *foliosquama* was collected on Barrow Island in 2010, and *A*. *apraefrontalis* was most recently collected from the Exmouth Gulf in 2004 and Broome in 2012; these records postdate both species’ disappearance from Ashmore and Hibernia where they were last sighted in 2001. Rediscoveries of presumed extinct reptiles are rare but usually involve longer intervals between disappearance and rediscovery. The Pygmy Blue-Tongue lizard [4] was rediscovered after 40 years, and the La Palma Giant Lizard and Terror Skink remained undetected in their known ranges for hundreds of years [55, 56]. The ‘false extinction’ of these lizards and the two *Aipysurus* sea snakes can, at least partially, be explained by the chronic lack of basic distributional and ecological data for most reptiles [1].

Molecular analysis of additional samples might resolve whether one or both *Aipysurus* species originated on the isolated Timor Sea reefs or dispersed there following a coastal origin. However, pairwise cytb distances between coastal and offshore lineages of 1% (*A*. *foliosquama*) and 0.7% (*A*. *apraefrontalis*) suggest recent connections, probably within the last few hundred thousand years (based on substitution rates estimated for cytochrome b in hydrophiines: [57]). Given the close molecular and morphological affinities of the coastal and offshore specimens we recommend that the newly recognised coastal populations remain in *A*. *foliosquama* and *A*. *apraefrontalis*. However, rapid speciation and post-speciation mitochondrial introgression have both been reported in sea snakes (e.g. [20]) and with further study might also be found here.

If *A*. *foliosquama* and *A*. *apraefrontalis* are indeed extinct at Ashmore and Hibernia, then the newly recognised coastal populations present another chance for these species’ survival. An urgent priority for conservation will be to assess the two species’ remaining distributions and population connectivity. The three coastal localities for *A*. *apraefrontalis* (Exmouth Gulf, ~600km to the north-north-east off Roebourne, and a further ~800km north-north-east off Broome: Fig. 1) suggest this species has a fairly large geographic range (extent of occurrence: [7]). However, the area of habitat it occupies within this range (area of occupancy:[7]) is likely to be small given that coral reefs are highly fragmented on the northwest Australian coast (Fig. 1). The two coastal *A*. *foliosquama* specimens were collected ~350km apart, but whether this species also has a wider distribution remains to be discovered. *Aipysurus foliosquama* is known only from heavily grown coral habitats (e.g. [11, 45]) so might have a smaller area of occupancy than *A*. *apraefrontalis*.

Effective recovery of the two species will further depend on threat distributions in their remaining range and the extent of their occurrence in protected areas. The northwest Commonwealth Marine Reserves Network includes 13 reserves [58] but large areas outside of these reserves are impacted by trawl-fishing, seismic surveys for oil and gas exploration, and destructive infrastructure developments. Of particular concern are mining and oil and gas projects that involve dredging and dumping marine sediment [59]. These activities are predicted to have areas of influence that can exceed 1000 square kilometres [59] potentially impacting protected reefs. A recent study linked dredging-induced sedimentation from the Gorgon natural gas project with coral stress and disease at Barrow Island and the nearby Montebello reefs [60]. Such threats to coral habitats will directly impact reef-specialists such *A*. *foliosquama*; one of the two coastal specimens of this species was beach-washed on the south coast of Barrow Island close to the Gorgon dredging and spoil disposal sites. Many other development projects are planned or underway in the Kimberley, Pilbara and mid-west coast (e.g. [59]). Hence, the inclusion of sea snakes in environmental impact studies for industry should be an immediate priority for marine conservation managers in northwest Australia.
